# Gender-Specific Risk Factors and Comorbidities of Bothersome Tinnitus

**DOI:** 10.3389/fnins.2020.00706

**Published:** 2020-09-23

**Authors:** Laura Basso, Benjamin Boecking, Petra Brueggemann, Nancy L. Pedersen, Barbara Canlon, Christopher R. Cederroth, Birgit Mazurek

**Affiliations:** ^1^Tinnitus Center, Charité – Universitätsmedizin Berlin, Berlin, Germany; ^2^Department of Medical Epidemiology and Biostatistics, Karolinska Institutet, Stockholm, Sweden; ^3^Laboratory of Experimental Audiology, Department of Physiology and Pharmacology, Karolinska Institutet, Stockholm, Sweden; ^4^National Institute for Health Research (NIHR) Nottingham Biomedical Research Centre, Nottingham University Hospitals NHS Trust, Nottingham, United Kingdom; ^5^Hearing Sciences, Division of Clinical Neuroscience, School of Medicine, University of Nottingham, Nottingham, United Kingdom

**Keywords:** tinnitus, gender difference, risk factors, cardiovascular disease, epilepsy, burnout, anxiety, education

## Abstract

**Objective:**

This study aims to identify gender-specific risk factors associated with the presence of bothersome tinnitus (compared with non-bothersome tinnitus), including sociodemographic and lifestyle factors, tinnitus-associated phenomena (hearing loss, traumatic experiences, sleep disturbances), and physical as well as mental comorbidities.

**Methods:**

We conducted a cross-sectional study using survey data from the Swedish LifeGene cohort containing information on self-reported tinnitus (*N* = 7615). We (1) analyzed risk factor and comorbidity frequencies, (2) computed multivariate logistic regression models to identify predictors of bothersome tinnitus within both genders, and (3) moderated logistic regression models to compare effects between genders.

**Results:**

(1) The majority of factors that differed in frequencies between bothersome and non-bothersome tinnitus were equal for both genders. Women with bothersome tinnitus specifically reported higher rates of cardiovascular disease, thyroid disease, epilepsy, fibromyalgia, and burnout, and men with bothersome tinnitus reported higher rates of alcohol consumption, Ménière’s disease, anxiety syndrome, and panic (compared with non-bothersome tinnitus, respectively). (2) Across both genders, multivariate logistic regression analyses revealed significant associations between bothersome tinnitus and age, reduced hearing ability, hearing-related difficulties in social situations, and reduced sleep quality. In women, bothersome tinnitus was specifically associated with cardiovascular disease and epilepsy; in men, with lower education levels and anxiety syndrome. (3) Moderated logistic regression analyses revealed that the effects of low education and anxiety syndrome were present in men, but not in women, whereas the effects of age, reduced hearing ability and related difficulties, cardiovascular disease, epilepsy, and burnout were not gender specific.

**Conclusion:**

Irrespective of gender, bothersome tinnitus is associated with higher age, reduced hearing ability, hearing-related difficulties, cardiovascular disease, epilepsy, and burnout. Gender-specific effects comprise low levels of education and the presence of anxiety syndrome for men. These findings need to be interpreted with caution, yet they suggest the presence of gender-specific biopsychosocial influences in the emergence or maintenance of bothersome tinnitus. Future studies ought to investigate the underlying mechanisms of the observed relationships.

## Introduction

Tinnitus is a highly prevalent symptom with 10–15% of adults being affected (Baguley et al., 2013). Many affected individuals perceive tinnitus as harmless and not debilitating (Henry et al., 2005). However, for 1% up to 7% of the population (Nondahl et al., 2002; Gallus et al., 2015; Ramage-Morin et al., 2019), tinnitus is a highly bothersome experience. In those individuals, the sound is generally perceived as an intrusive threat, leading to emotional distress (Henry et al., 2005; Cima, 2018).

Epidemiological research on tinnitus is mixed. While most studies have cross-sectional designs with inherent limitations, a handful of longitudinal studies has contributed to identifying risk factors for tinnitus. The most clearly identified risk factor is hearing loss (Nondahl et al., 2002; Gopinath et al., 2010; Aarhus et al., 2015; Bogo et al., 2017). Symptoms of temporomandibular disorders (Bernhardt et al., 2011; Lee et al., 2016) and smoking were found to be associated with an increased risk of tinnitus (Nondahl et al., 2010), whereas higher caffeine intake (Glicksman et al., 2014) and moderate alcohol consumption were associated with a lower risk (Nondahl et al., 2010). Cross-sectional studies further suggest associations between tinnitus and increased age (Shargorodsky et al., 2010; Park et al., 2014; Gallus et al., 2015; Kim et al., 2015), sleep disturbances (Axelsson and Ringdahl, 1989; Izuhara et al., 2013), and sociodemographic factors (Unterrainer et al., 2001; Hoekstra et al., 2014; Gallus et al., 2015; Kim et al., 2015).

Tinnitus can be associated with a range of physical and mental conditions (Baguley et al., 2013), some of which appear relevant for bothersome tinnitus (Basso et al., submitted). Many of these conditions, like cardiovascular diseases (Regitz-Zagrosek and Kararigas, 2017), chronic musculoskeletal pain (Wijnhoven et al., 2006), thyroid diseases (Vanderpump et al., 1995), Ménière’s disease (Smith et al., 2019), depression (Salk et al., 2017), and anxiety disorders (Bandelow and Michaelis, 2015) are marked by gender differences.

Regarding bothersome tinnitus, relationships with stress were found in cross-sectional studies (Park et al., 2014; Kim et al., 2015). Moreover, adverse life events can contribute to tinnitus aggravation (Zeng et al., 2016) and bidirectional links between symptoms of posttraumatic stress disorder (PTSD) and tinnitus severity are known (Hinton et al., 2006; Fagelson, 2007).

Whether sex or gender impacts on tinnitus severity is poorly understood. For instance, in some studies, women were found to exhibit higher levels of tinnitus-related distress or annoyance (Seydel et al., 2013; Gallus et al., 2015; Schlee et al., 2017), but opposite findings exist as well (Jalessi et al., 2013), and some studies found no severity differences between men and women (Axelsson and Ringdahl, 1989; Gopinath et al., 2010; Hoekstra et al., 2014). Moreover, a recent study found an association between tinnitus severity and suicide attempts in women only (Lugo et al., 2019), highlighting the importance to investigate gender differences in severe tinnitus. Differences in risk factors for bothersome tinnitus between men and women have not yet been investigated.

Given the paucity of research using sex as a biological variable (SABV), the aim of the present study is to investigate gender differences in risk factors for bothersome tinnitus in a large general population sample, covering sociodemographic factors, lifestyle factors, hearing loss, traumatic experiences, sleep disturbances, and physical and mental comorbidities. Logistic regression models are used to identify risk factors within both genders, and moderation models are used to assess whether the effects of the respective factors on bothersome tinnitus are moderated by gender, that is, are different for women and men.

## Materials and Methods

### Data Source and Study Design

The sample of this study was drawn from the LifeGene cohort. LifeGene is a population-based study conducted in Sweden (Almqvist et al., 2011; LifeGene, 2017). Cross-sectional data of the web-based LifeGene survey collected between 2009 and 2016 were used. Participants were recruited via random selection, invitation by other participants, and self-registration (Almqvist et al., 2011; LifeGene, 2017). From the 31926 participants who completed the survey, all individuals with self-reported tinnitus [*N* = 7615 (23.9%)] were included in the present study. The same sample was used in Basso et al. (submitted). All participants provided informed consent (for participants under the age of 18, informed consent was provided by the participants’ legal guardian/next of kin). The project has been approved by the local ethics committee “*Regionala etikprövningsnämnden*” in Stockholm (2015/2129-31/1).

### Variables

The investigated risk factors were grouped into (1) sociodemographic and lifestyle factors (age, marital status, education level, employment, alcohol use, smoking status, snus and drug use), (2) tinnitus-associated phenomena (hearing ability and hearing-related difficulties in social situations, sleep quality and sleep disturbances, and traumatic/stressful experiences), and (3) physical and (4) mental comorbidities; see Supplementary Figure S1. Information on sociodemographic factors was selected from the “socio-demography” module of the LifeGene survey, information on lifestyle factors from the “living habits” module, information on comorbidities (past or present), sleep quality/disturbances, hearing ability and hearing-related difficulties in social situations from the “medical history” module, and information regarding traumatic/stressful life events from the “mental health” module. Participants’ experience of the tinnitus [“Is there a constant ringing in the ears or do you have any other bothersome sound in the ears (tinnitus)?”] as constant and bothersome (“All the time, the sound is very bothersome”) or intermittent and non-bothersome (“Sometimes, but the sound doesn’t bother me”) was defined as the dependent variable in all analyses.

Sociodemographic factors included age, marital status (married, cohabiting, single, separated/divorced, living apart, widowed, same-sex marriage), highest or current level of education (9-year primary school, secondary school, university, other), and employment [employed, unemployed, running an owned or part-owned company, age pension, activity or sickness benefit (early retirement) due to illness/disability, sick leave (for 2 months or longer), parental leave (for 2 months or longer), student, on leave, housewife/man, other]. Lifestyle factors included smoking status and the use of alcohol, snus, and illicit drugs. Alcohol consumption was assessed by the question: “If you consider the last 12 months, how often have you been drinking alcohol?” (“four times a week or more,” “2–3 times per week,” “once a week,” “2–3 times per month,” “once a month or less,” “never”). Smoking status was categorized as “current smoker” (>100 cigarettes in life, at least 1 cigarette/day when smoking most AND currently smoking), “ex-smoker” (>100 cigarettes in life, at least 1 cigarette/day when smoking most, NOT currently smoking), and “non-smoker” (never or occasional: <100 cigarettes in life OR less than 1 cigarette/day when smoking most). The use of snus, a smokeless tobacco product (moist powder) usually placed under the upper lip, was categorized as “current snus user” (>5 boxes in life, 1 box lasting <1 week when using it most AND present use), “ex-user” (>5 boxes in life, 1 box lasting <1 week when using it most, NO present use), “non-user” (never, <5 boxes in life, OR 1 box lasting >1 week when using it most). The use of illicit drugs or unprescribed medication was categorized as “current drug user” (tried more than once AND present use), “ex-user” (tried more than once, NO present use), “non-user” (never OR not more than once) with regard to the following substances: (1) cannabis, marijuana, hash; (2) amphetamine; (3) cocaine; (4) sobril, oxascand, stesolid, diazepam, xanor, alprazolam; (5) stilnoct, zolpidem, imovane, zopiclone; (6) growth hormone; (7) anabolic steroids; (8) codeine, citodon, treo comp, panocod; (9) tramadol, tradolan, tiparol, nobligan; (10) heroin; (11) opium; (12) hallucinogens (psilocybin, psilocin); (13) LSD; (14) ecstasy; (15) GHB; (16) methylphenidate (ritalin, concerta); (17) morphine; (18) subutex, suboxone; or (19) other drug or medication.

Tinnitus-associated phenomena included hearing ability, hearing-related difficulties in social situations, sleep quality, sleep disturbances, and traumatic/stressful experiences. Hearing ability was assessed by the question “How is your hearing?” (“good,” “somewhat reduced,” “very reduced”). Hearing-related difficulties in social situations were assessed by combining the following questions into a mean variable: “Do you have difficulties hearing when speaking to one person in a silent room?,” “Do you have difficulties hearing when speaking to multiple people at the same time?,” “Do you have difficulties hearing when speaking to someone in city traffic?,” “Do you have difficulties hearing where different sounds come from, e.g., cars in traffic?” and “Do you have problems with your hearing and are therefore avoiding meeting people?” (3 = “yes, very difficult,” 2 = “sometimes, a little difficult,” 1 = “no, not at all”). Sleep quality was assessed by the question “How do you sleep usually?” (response scale ranging from 1 = “very bad” to 5 = “very good”; for the analyses, the scale was inverted so that higher values reflect poorer sleep quality). In addition, participants were asked to rate how problematic their sleep disturbances are (“To what degree are sleep disturbances a problem in your life?”), with the response scale ranging from 1 = “no problem at all” to 5 = “a big problem.” Traumatic/stressful life events were assessed by calculating the sum of reported traumatic/stressful life events experienced in childhood or adulthood, see Supplementary Table S1 for all 32 items.

The following physical comorbidities were selected because of their proposed association with tinnitus in the literature: hypertension, hyperlipidemia, cardiovascular disease (angina, myocardial infarction, or cardiac arrhythmia), asthma, diabetes, thyroid disease, chronic shoulder pain, osteoarthritis, rheumatoid arthritis, systemic lupus erythematosus, migraine, Ménière’s disease, epilepsy, multiple sclerosis, and fibromyalgia. The following mental comorbidities were included: burnout, depression, bipolar disease, (generalized) anxiety syndrome, panic, agoraphobia, social anxiety/phobia, obsessive-compulsive disorder, and PTSD. The survey questions assessing these conditions asked for their past or present occurrence.

### Statistical Analysis

Statistical analyses were computed with IBM SPSS Statistics (version 25) for Windows 7. The significance level was set to α = 0.05. In the first step, we identified risk factors for bothersome (vs. non-bothersome) tinnitus within each gender: (1) by comparing frequencies and medians of each variable between non-bothersome and bothersome tinnitus for women and men separately and (2) by further analyzing relevant variables [identified in (1)] as predictors of bothersome tinnitus in logistic regression analyses for women and men separately. Lastly, (3) for the comparison between genders, we tested whether gender moderated the effects of each risk factor [identified in (2)] across the whole sample.

(1) Pearson’s *X ^2^* tests were conducted to compare the frequencies of categorical variables between non-bothersome and bothersome tinnitus for both genders separately. Continuity correction was used for 2 × 2 tables, and adjusted residuals (ARs) were calculated to compare between the frequencies of categories (ARs ≥ 1.96 or ≤−1.96 indicate significant differences). For continuous variables, Mann–Whitney *U* tests were used because of non-normally distributed data. (2) To test the unique association of relevant variables with bothersome tinnitus (identified by previous analyses), multivariate logistic regression models were calculated for women and men separately including the following predictors: Model 1: sociodemographic and lifestyle factors, Model 2: tinnitus-associated phenomena, Model 3: physical comorbidities, Model 4: mental comorbidities, and Model 5: all significant predictors from Models 1 to 4. The assumptions of logistic regression were met (no multicollinearity was present among predictors, all variance inflation factor (VIF) values <2, and the Box-Tidwell approach showed a linear relationship between the continuous predictor age and the logit of the outcome). Regarding outliers, 150 female participants (3.5%) and 123 male participants (3.7%) had studentized residuals greater than 2 in Model 5, which were kept in the analyses; no cases had studentized residuals greater than 3. For model evaluation, Nagelkerke *R*^2^ is reported as a measure of goodness-of-fit. (3) To test whether the relationships between the predictors included in Model 5 and bothersome tinnitus are different depending on gender, separate moderation models by gender were conducted for each predictor by using the PROCESS syntax version 3.4 by Hayes (2018). For all odds ratios (OR), 95% confidence intervals were computed.

Completeness of the data was high; in total, 1.5% of values were missing. The response rate was lowest with 83.8% on hearing-related difficulties in social situations, followed by 88.3% on employment; other response rates varied between 96.7 and 98.9% on eight variables (marital status, education, alcohol consumption, smoking status, snus use, drug use, hearing ability, traumatic experiences), and between 99.5 and 99.7% on 26 variables (sleep quality, sleep disturbances, and all comorbidities). All data was available for age and gender.

## Results

### Sample Description

**FIGURE 1:**
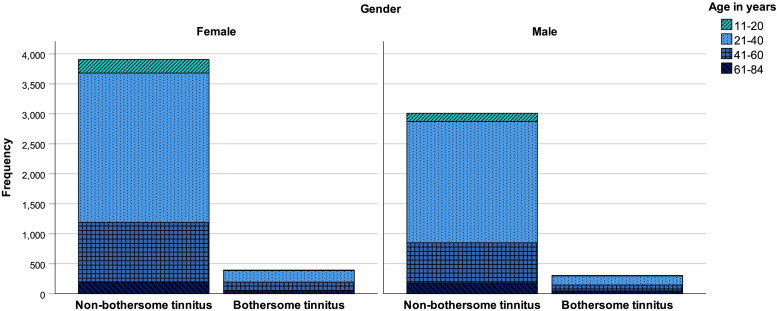
Histogram of non-bothersome and bothersome tinnitus (count data) by age and gender.

Of the 7615 participants with tinnitus, 697 reported bothersome tinnitus (9.2%). Females represented 56.5% of the sample (4301 participants): 393 with bothersome tinnitus (9.1%). In males (3314 participants), 304 reported bothersome tinnitus (9.2%). Participants were between 11 and 84 years old (*M* = 35.80 years, SD = 12.44 years); on average, female participants were 35.62 years old (SD = 12.45 years) and male participants were 36.03 years old (SD = 12.42 years); *U* = 6931148, *p* = 0.040. The composition of the sample by age and gender for non-bothersome and bothersome tinnitus is displayed in Figure 1. Most frequently, participants were cohabiting (33.4%), married (25.0%), or single (24.0%); education (highest or current) was mostly at university level (61.7%); and most participants were employed (53.7%) or students (15.0%).

### Gender Differences Between Bothersome and Non-bothersome Tinnitus

#### Differences in Frequencies/Medians

##### Sociodemographic factors

Female participants with bothersome tinnitus were significantly older than those with non-bothersome tinnitus, *U* = 598970, *p* < 0.001; they differed in marital status, *X ^2^*(6) = 20.82, *p* = 0.002 (more often married, less often cohabiting, less often single, more often separated or divorced), in their highest or current level of education, **X*^*2*^*(3) = 18.69, *p* < 0.001 (less often university, more often “other”), as well as in their present employment situation, **X*^*2*^*(10) = 60.60, *p* < 0.001 (less often employed, more often unemployed, more often in age pension, and more often in early retirement due to illness/disability); see Table 1.

**TABLE 1:** Female participants: Differences in sociodemographic, lifestyle factors, and tinnitus-associated phenomena between bothersome and non-bothersome tinnitus.

	**Non-bothersome tinnitus**		**Bothersome tinnitus**	
**Variable**	**Frequency (count data)**	**Adjusted residuals**	**Frequency (count data)**	**Adjusted residuals**

**Marital status^∗∗^**	*n* = 3781		*n* = 386	

**Married**	25.7% (971)	−3.0	32.6% (126)	3.0
**Cohabiting**	33.7% (1274)	2.0	28.8% (111)	−2.0
**Single**	24.3% (918)	2.4	18.9% (73)	−2.4
**Separated/divorced**	5.6% (210)	−2.8	9.1% (35)	2.8
Living apart	10.2% (386)	0.1	10.1% (39)	−0.1
Widowed	0.5% (20)	0.0	0.5% (2)	0.0
Same-sex marriage	0.1% (2)	0.5	0.0% (0)	−0.5

**Education^∗∗∗^**	*n* = 3807		*n* = 386	

Nine-year primary school	2.2% (83)	−1.8	3.6% (14)	1.8
Secondary school	23.1% (880)	−0.8	24.9% (96)	0.8
**University**	66.1% (2516)	3.4	57.5% (222)	−3.4
**Other**	8.6% (328)	−3.5	14.0% (54)	3.5
**Employment^∗∗∗^**	*n* = 3416		*n* = 356	
**Employed**	60.6% (2069)	2.4	53.9% (192)	−2.4
**Unemployed**	3.2% (108)	−3.0	6.2% (22)	3.0
Running an owned or part-owned company	6.6% (225)	0.9	5.3% (19)	−0.9
**Age pension**	3.0% (103)	−6.0	9.3% (33)	6.0
**Activity or sickness benefit (early retirement) due to illness/disability**	1.0% (34)	−3.0	2.8% (10)	3.0
Sick leave (for 2 months or longer)	1.2% (42)	0.2	1.1% (4)	−0.2
Parental leave (for 2 months or longer)	3.6% (123)	0.2	3.4% (12)	−0.2
Student	18.6% (636)	1.6	15.2% (54)	−1.6
On leave	0.1% (3)	0.6	0.0% (0)	−0.6
Housewife/man	0.3% (9)	1.0	0.0% (0)	−1.0
Other	1.9% (64)	−1.2	2.8% (10)	1.2

**Smoking status^∗^**	*n* = 3811		*n* = 384	

Non-smoker	60.9% (2321)	1.4	57.3% (220)	−1.4
**Ex-smoker**	28.7% (1095)	−2.6	35.2% (135)	2.6
Smoker	10.4% (395)	1.7	7.6% (29)	−1.7
**Hearing ability^∗∗∗^**	*n* = 3780		*n* = 377	
**Good**	70.4% (2663)	12.1	39.8% (150)	−12.1
**Somewhat reduced**	28.4% (1073)	−9.7	52.5% (198)	9.7
**Very reduced**	1.2% (44)	−9.2	7.7% (29)	9.2

	***n***	**Median**	***n***	**Median**

**Age^∗∗∗^**	3908	32	393	40
**Hearing-related difficulties in social situations^∗∗∗^**	3245	1.4	356	1.5
**Traumatic/stressful experiences^∗∗∗^**	3843	3	388	4
**Poor sleep quality^∗∗∗^**	3898	2	393	3
**Problematic sleep disturbances^∗∗∗^**	3895	2	393	3

*Factors/categories in bold font indicate significant differences in frequencies (adjusted residuals ≥1.96 or ≤−1.96). ^∗∗∗^*p* < 0.001, ^∗∗^*p* < 0.01, ^∗^*p* < 0.05.*

Male participants with bothersome tinnitus were significantly older than those with non-bothersome tinnitus, *U* = 341380, *p* < 0.001; they differed in marital status, **X*^*2*^*(6) = 40.12, *p* < 0.001 (more often married, less often single, and more often widowed), in their highest or current level of education, **X*^*2*^*(3) = 49.05, *p* < 0.001 (more often primary school, more often secondary school, and less often university), as well as in their present employment situation, *X*^*2*^(10) = 35.51, *p* < 0.001 (more often running an owned or part-owned company, more often in age pension, less often student, and more often “other”); see Table 2.

**TABLE 2:** Male participants: Differences in sociodemographic, lifestyle factors, and tinnitus-associated phenomena between bothersome and non-bothersome tinnitus.

	**Non-bothersome tinnitus**		**Bothersome tinnitus**	
**Variable**	**Frequency (count data)**	**Adjusted residuals**	**Frequency (count data)**	**Adjusted residuals**
**Marital status^∗∗∗^**	*n* = 2915		*n* = 299	

**Married**	23.9% (696)	−5.0	37.1% (111)	5.0
Cohabiting	36.5% (1063)	1.5	32.1% (96)	−1.5
**Single**	26.6% (776)	2.8	19.1% (57)	−2.8
Separated/divorced	3.5% (102)	−0.5	4.0% (12)	0.5
Living apart	9.5% (276)	1.4	7.0% (21)	−1.4
**Widowed**	0.0% (1)	−3.4	0.7% (2)	3.4
Same-sex marriage	0.0% (1)	0.3	0.0% (0)	−0.3

**Education^∗∗∗^**	*n* = 2944		*n* = 301	

**Nine-year primary school**	2.7% (80)	−6.1	9.3% (28)	6.1
**Secondary school**	27.9% (820)	−2.0	33.2% (100)	2.0
**University**	61.7% (1815)	4.8	47.5% (143)	−4.8
Other	7.8% (229)	−1.3	10.0% (30)	1.3

**Employment^∗∗∗^**	*n* = 2679		*n* = 275	

Employed	62.4% (1671)	1.6	57.5% (158)	−1.6
Unemployed	3.2% (86)	−0.1	3.3% (9)	0.1
**Running an owned or part-owned company**	11.4% (305)	−2.1	15.6% (43)	2.1
**Age pension**	3.8% (101)	−4.7	9.8% (27)	4.7
Activity or sickness benefit (early retirement) due to illness/disability	0.4% (11)	−0.8	0.7% (2)	0.8
Sick leave (for 2 months or longer)	0.6% (15)	−0.3	0.7% (2)	0.3
Parental leave (for 2 months or longer)	1.0% (28)	0.5	0.7% (2)	−0.5
**Student**	15.9% (425)	3.0	9.1% (25)	−3.0
On leave	0.3% (7)	0.8	0.0% (0)	−0.8
Housewife/man	0.0% (1)	0.3	0.0% (0)	−0.3
**Other**	1.1% (29)	−2.1	2.5% (7)	2.1

**Alcohol^∗∗^**	*n* = 2957		*n* = 298	

**4 times a week or more**	5.5% (163)	−2.5	9.1% (27)	2.5
2–3 times a week	26.4% (781)	0.3	25.5% (76)	−0.3
Once a week	21.5% (637)	1.4	18.1% (54)	−1.4
2–3 times a month	26.1% (772)	1.4	22.5% (67)	−1.4
Once a month or less	17.7% (522)	−0.6	19.1% (57)	0.6
**Never**	2.8% (82)	−2.8	5.7% (17)	2.8

**Smoking status^∗∗^**	*n* = 2952		*n* = 299	

**Non-smoker**	64.5% (1903)	3.5	54.2% (162)	−3.5
**Ex-smoker**	28.5% (842)	−3.2	37.5% (112)	3.2
Smoker	7.0% (207)	−0.9	8.4% (25)	0.9

**Hearing ability^∗∗∗^**	*n* = 2974		*n* = 299	

**Good**	66.1% (1966)	11.8	31.4% (94)	−11.8
**Somewhat reduced**	32.9% (977)	−9.5	60.5% (181)	9.5
**Very reduced**	1.0% (31)	−9.0	8.0% (24)	9.0

	***n***	**Median**	***n***	**Median**

**Age^∗∗∗^**	3010	33	304	39.5
**Hearing-related difficulties in social situations^∗∗∗^**	2497	1.2	281	1.4
**Traumatic/stressful experiences^∗∗^**	2966	3	299	3
**Poor sleep quality^∗∗∗^**	2997	2	304	3
**Problematic sleep disturbances^∗∗∗^**	2996	2	304	2

*Factors/categories in bold font indicate significant differences in frequencies (adjusted residuals ≥1.96 or ≤−1.96). ^∗∗∗^*p* < 0.001, ^∗∗^*p* < 0.01, ^∗^*p* < 0.05.*

##### Lifestyle factors

Compared with women with non-bothersome tinnitus, women with bothersome tinnitus were more often ex-smokers, **X*^*2*^*(2) = 8.39, *p* = 0.015. Men with bothersome tinnitus were more often ex-smokers and less often non-smokers than male participants with non-bothersome tinnitus, **X*^*2*^*(2) = 12.60, *p* = 0.002, and in addition, they reported more often drinking alcohol four times a week and never than those with non-bothersome tinnitus, **X*^*2*^*(5) = 16.78, *p* = 0.005. Other lifestyle variables did not differ; see Table 1 for women and Table 2 for men.

##### Tinnitus-associated phenomena

For both women and men, participants with bothersome tinnitus reported more often somewhat reduced or very reduced hearing ability compared with participants with non-bothersome tinnitus [women: **X*^*2*^*(2) = 196.13, *p* < 0.001; men: **X*^*2*^*(2) = 189.60, *p* < 0.001], as well as more hearing-related difficulties in social situations (women: *U* = 388097, *p* < 0.001; men: *U* = 243912, *p* < 0.001), more traumatic/stressful experiences (women: *U* = 653215, *p* < 0.001; men: *U* = 391945, *p* = 0.001), poorer sleep quality (women: *U* = 649596, *p* < 0.001; men: *U* = 349794, *p* < 0.001), and more problematic sleep disturbances (women: *U* = 659704, *p* < 0.001; men: *U* = 355895, *p* < 0.001); see Tables 1, 2.

##### Physical and mental comorbidities

Women with bothersome tinnitus reported higher rates of hypertension, **X*^*2*^*(1) = 4.98, *p* = 0.026, hyperlipidemia, **X*^*2*^*(1) = 8.19, *p* = 0.004, cardiovascular disease, **X*^*2*^*(1) = 18.40, *p* < 0.001, thyroid disease, **X*^*2*^*(1) = 13.82, *p* < 0.001, chronic shoulder pain, **X*^*2*^*(1) = 21.68, *p* < 0.001, osteoarthritis, **X*^*2*^*(1) = 18.41, *p* < 0.001, epilepsy, **X*^*2*^*(1) = 6.87, *p* = 0.009, and fibromyalgia, **X*^*2*^*(1) = 16.56, *p* < 0.001, than women with non-bothersome tinnitus, as well as higher rates of burnout, **X*^*2*^*(1) = 17.62, *p* < 0.001, depression, **X*^*2*^*(1) = 7.93, *p* = 0.005, and social anxiety, **X*^*2*^*(1) = 5.06, *p* = 0.025; see Table 3.

**TABLE 3:** Differences in physical and mental comorbidities between bothersome and non-bothersome tinnitus.

	**Non-bothersome tinnitus**		**Bothersome tinnitus**	
**Variable**	**Frequency (count data)**	**Adjusted residuals**	**Frequency (count data)**	**Adjusted residuals**
	**Female participants**			

	*n* = 3894		*n* = 392	
**Hypertension^∗^**	5.7% (223)	−2.3	8.7% (34)	2.3
**Hyperlipidemia^∗∗^**	2.5% (97)	−3.0	5.1% (20)	3.0
**Cardiovascular disease^∗∗∗^**	4.2% (165)	−4.4	9.2% (36)	4.4
Asthma	12.5% (485)	0.0	12.5% (49)	0.0
Diabetes	0.5% (19)	−1.4	1.0% (4)	1.4
**Thyroid disease^∗∗∗^**	5.8% (226)	−3.8	10.7% (42)	3.8
	*n* = 3891		*n* = 392	
**Chronic shoulder pain^∗∗∗^**	7.2% (282)	−4.8	14.0% (55)	4.8
**Osteoarthritis^∗∗∗^**	5.4% (212)	−4.4	11.0% (43)	4.4
Rheumatoid arthritis	0.8% (30)	0.0	0.8% (3)	0.0
Systemic lupus erythematosus	0.1% (5)	0.7	0.0% (0)	−0.7
Migraine	19.2% (749)	−1.2	21.7% (85)	1.2
Ménière’s disease	0.2% (8)	−1.2	0.5% (2)	1.2
**Epilepsy^∗∗^**	0.7% (26)	−2.9	2.0% (8)	2.9
Multiple sclerosis	0.1% (5)	0.7	0.0% (0)	−0.7
**Fibromyalgia^∗∗∗^**	1.3% (50)	−4.3	4.1% (16)	4.3
**Burnout^∗∗∗^**	12.1% (470)	−4.3	19.6% (77)	4.3
**Depression^∗∗^**	25.0% (972)	−2.9	31.6% (124)	2.9
Bipolar disease	0.7% (29)	−1.6	1.5% (6)	1.6
Anxiety syndrome	12.3% (478)	−1.6	15.1% (59)	1.6
Panic	13.9% (542)	−0.3	14.5% (57)	0.3
Agoraphobia	0.7% (28)	−1.2	1.3% (5)	1.2
**Social anxiety^∗^**	3.7% (143)	−2.4	6.1% (24)	2.4
Obsessive-compulsive disorder	2.5% (97)	−0.7	3.1% (12)	0.7
Posttraumatic stress disorder	2.2% (84)	−1.8	3.6% (14)	1.8

	**Male participants**			

	*n* = 2992		*n* = 303	
**Hypertension^∗∗^**	5.9% (176)	−3.2	10.6% (32)	3.2
**Hyperlipidemia^∗∗^**	3.8% (114)	−3.4	7.9% (24)	3.4
Cardiovascular disease	3.7% (112)	−1.9	5.9% (18)	1.9
Asthma	9.7% (289)	−0.9	11.2% (34)	0.9
Diabetes	0.7% (22)	1.5	0.0% (0)	−1.5
Thyroid disease	0.8% (25)	−1.4	1.7% (5)	1.4
	*n* = 2991		*n* = 303	
**Chronic shoulder pain^∗∗∗^**	2.8% (85)	−5.0	8.3% (25)	5.0
**Osteoarthritis^∗∗^**	3.7% (111)	−3.0	7.3% (22)	3.0
Rheumatoid arthritis	0.3% (9)	−1.0	0.7% (2)	1.0
Systemic lupus erythematosus	0.0% (0)	0.0	0.0% (0)	0.0
Migraine	8.9% (267)	−0.8	10.2% (31)	0.8
**Ménière’s disease^∗∗∗^**	0.2% (5)	−4.5	1.7% (5)	4.5
Epilepsy	0.7% (21)	0.8	0.3% (1)	−0.8
Multiple sclerosis	0.2% (5)	0.7	0.0% (0)	−0.7
Fibromyalgia	0.1% (4)	0.6	0.0% (0)	−0.6
Burnout	7.6% (227)	−1.0	9.2% (28)	1.0
**Depression^∗∗^**	14.5% (433)	−2.8	20.5% (62)	2.8
Bipolar disease	0.8% (23)	0.9	0.3% (1)	−0.9

	**Non-bothersome tinnitus**		**Bothersome tinnitus**	
**Variable**	**Frequency (count data)**	**Adjusted residuals**	**Frequency (count data)**	**Adjusted residuals**

	**Female participants**			

**Anxiety syndrome^∗∗∗^**	7.0% (210)	−4.1	13.5% (41)	4.1
**Panic^∗∗^**	7.4% (221)	−3.6	13.2% (40)	3.6
Agoraphobia	0.3% (10)	−0.9	0.7% (2)	0.9
**Social anxiety^∗∗^**	2.9% (88)	−3.4	6.6% (20)	3.4
Obsessive-compulsive disorder	1.4% (43)	−1.2	2.3% (7)	1.2
Posttraumatic stress disorder	0.8% (23)	−1.6	1.7% (5)	1.6

*Factors in bold font indicate significant differences in frequencies (adjusted residuals ≥1.96 or ≤−1.96). ^∗∗∗^*p* < 0.001, ^∗∗^*p* < 0.01, ^∗^*p* < 0.05.*

Men with bothersome tinnitus reported higher rates of hypertension, **X*^*2*^*(1) = 9.41, *p* = 0.002, hyperlipidemia, **X*^*2*^*(1) = 10.58, *p* = 0.001, chronic shoulder pain, **X*^*2*^*(1) = 23.29, *p* < 0.001, osteoarthritis, **X*^*2*^*(1) = 8.05, *p* = 0.005, and Ménière’s disease, **X*^*2*^*(1) = 15.39, *p* < 0.001, than men with non-bothersome tinnitus, as well as higher rates of depression, *X*^2^(1) = 7.26, *p* = 0.007, anxiety syndrome, **X*^*2*^*(1) = 15.65, *p* < 0.001, panic, **X*^*2*^*(1) = 11.96, *p* = 0.001, and social anxiety, **X*^*2*^*(1) = 10.49, *p* = 0.001; see Table 3.

#### Logistic Regression Analysis (Comparison Within Genders)

##### Female participants

###### Model 1: Sociodemographic and lifestyle factors

Higher age, level of education (all factor levels non-significant), and employment status (being unemployed, in early retirement due to illness/disability, and student; contrasted with being employed) significantly predicted bothersome tinnitus, Nagelkerke *R*^2^ = 0.050. Marital status and smoking status showed no effect. See Table 4, Model 1.

**TABLE 4:** Female participants: Logistic regression models for sociodemographic and lifestyle factors (Model 1), tinnitus-associated phenomena (Model 2), physical comorbidities (Model 3), and mental comorbidities (Model 4).

						**95% CI**	
**Variable**	**β**	**SE β**	**Wald’s *X ^2^***	***p***	**OR**	**Lower**	**Upper**
**Model 1: sociodemographic and lifestyle factors**							
Age^∗∗∗^	0.027	0.007	17.41	<0.001	1.028	1.015	1.041
Marital status							
Education^∗^			8.92	0.030			
Employment^∗^			22.13	0.014			
Unemployed^∗∗^	0.860	0.256	11.25	0.001	2.364	1.430	3.908
Early retirement due to illness/disability^∗^	0.775	0.394	3.87	0.049	2.172	1.003	4.704
Student^∗^	0.370	0.186	3.97	0.046	1.447	1.006	2.082
Smoking status							
**Model 2: tinnitus-associated phenomena**							
Hearing ability^∗∗∗^			47.52	<0.001			
Somewhat reduced^∗∗∗^	0.853	0.133	41.38	<0.001	2.347	1.810	3.043
Very reduced^∗∗∗^	1.438	0.304	22.43	<0.001	4.213	2.323	7.640
Hearing-related difficulties in social situations^∗∗∗^	1.023	0.165	38.27	<0.001	2.782	2.012	3.848
Traumatic/stressful experiences							
Poor sleep quality^∗∗^	0.206	0.074	7.76	0.005	1.229	1.063	1.420
Problematic sleep disturbances							
**Model 3: physical comorbidities**							
Hypertension							
Hyperlipidemia							
Cardiovascular disease^∗∗^	0.658	0.198	11.09	0.001	1.931	1.311	2.844
Thyroid disease^∗∗^	0.536	0.182	8.70	0.003	1.708	1.197	2.439
Chronic shoulder pain^∗∗^	0.506	0.167	9.15	0.002	1.659	1.195	2.304
Osteoarthritis^∗∗^	0.550	0.184	8.92	0.003	1.733	1.208	2.487
Epilepsy^∗^	1.041	0.415	6.31	0.012	2.833	1.257	6.383
Fibromyalgia^∗^	0.759	0.312	5.94	0.015	2.137	1.160	3.937
**Model 4: mental comorbidities**							
Burnout^∗∗^	0.488	0.148	10.94	0.001	1.629	1.220	2.176
Depression							
Social anxiety							

*Only significant results are displayed. OR, odds ratio. The coefficients of employment are contrasted with being employed; those of self-rated hearing ability are contrasted with good hearing. ^∗∗∗^*p* < 0.001, ^∗∗^*p* < 0.01, ^∗^*p* < 0.05.*

###### Model 2: Tinnitus-associated phenomena

Hearing ability (somewhat reduced hearing ability and very reduced hearing ability; contrasted with good hearing), hearing-related difficulties in social situations, and poor sleep quality significantly predicted bothersome tinnitus, Nagelkerke *R^2^* = 0.117. Traumatic experiences and sleep disturbances showed no effect. See Table 4, Model 2.

###### Model 3: Physical comorbidities

The past or present occurrence of cardiovascular disease, thyroid disease, chronic shoulder pain, osteoarthritis, epilepsy, and fibromyalgia significantly predicted bothersome tinnitus, Nagelkerke *R*^2^ = 0.035. Hypertension and hyperlipidemia showed no influence. See Table 4, Model 3.

###### Model 4: Mental comorbidities

The past or present occurrence of burnout significantly predicted bothersome tinnitus, Nagelkerke *R^2^* = 0.011. Depression and social anxiety showed no influence. See Table 4, Model 4.

##### Male participants

###### Model 1: Sociodemographic and lifestyle factors

Higher age and level of education (secondary school, university, and “other”; contrasted with primary school) significantly predicted bothersome tinnitus, Nagelkerke *R*^2^ = 0.078. Marital status, employment, alcohol consumption, and smoking status showed no influence. See Table 5, Model 1.

**TABLE 5:** Male participants: Logistic regression models for sociodemographic and lifestyle factors (Model 1), tinnitus-associated phenomena (Model 2), physical comorbidities (Model 3), and mental comorbidities (Model 4).

						**95% CI**	
**Variable**	**β**	**SE β**	**Wald’s *X ^2^***	***p***	**OR**	**Lower**	**Upper**
**Model 1: sociodemographic and lifestyle factors**							
Age^∗∗∗^	0.032	0.007	18.57	<0.001	1.033	1.018	1.048
Marital status							
Education^∗∗∗^			21.08	<0.001			
Secondary school^∗∗^	−0.878	0.272	10.44	0.001	0.416	0.244	0.708
University^∗∗∗^	−1.193	0.266	20.11	<0.001	0.303	0.180	0.511
Other^∗∗^	−0.891	0.321	7.69	0.006	0.410	0.219	0.770
Employment							
Alcohol							
Smoking status							
**Model 2: tinnitus-associated phenomena**							
Hearing ability^∗∗∗^			68.13	<0.001			
Somewhat reduced^∗∗∗^	1.130	0.153	54.90	<0.001	3.097	2.296	4.176
Very reduced^∗∗∗^	2.125	0.340	39.02	<0.001	8.372	4.298	16.308
Hearing-related difficulties in social situations^∗∗∗^	0.799	0.207	14.93	<0.001	2.224	1.483	3.336
Traumatic/stressful experiences							
Poor sleep quality^∗∗^	0.226	0.085	7.01	0.008	1.253	1.060	1.481
Problematic sleep disturbances							
**Model 3: physical comorbidities**							
Hypertension							
Hyperlipidemia							
Chronic shoulder pain^∗∗∗^	0.992	0.243	16.71	<0.001	2.697	1.676	4.339
Osteoarthritis							
Ménière’s disease^∗∗^	2.084	0.658	10.02	0.002	8.037	2.211	29.211
**Model 4: mental comorbidities**							
Depression							
Anxiety syndrome^∗^	0.449	0.224	4.00	0.045	1.566	1.009	2.430
Panic							
Social anxiety							

*Only significant results are displayed. OR, odds ratio. The coefficients of education are contrasted with 9-year primary school; those of self-rated hearing ability are contrasted with good hearing. ^∗∗∗^*p* < 0.001, ^∗∗^*p* < 0.01, ^∗^*p* < 0.05.*

###### Model 2: Tinnitus-associated phenomena

Hearing ability (somewhat reduced hearing ability and very reduced hearing ability; contrasted with good hearing), hearing-related difficulties in social situations, and poor sleep quality significantly predicted bothersome tinnitus, Nagelkerke *R^2^* = 0.138. Traumatic experiences and sleep disturbances were not associated with bothersome tinnitus. See Table 5, Model 2.

###### Model 3: Physical comorbidities

The past or present occurrence of chronic shoulder pain and Ménière’s disease significantly predicted bothersome tinnitus, Nagelkerke *R^2^* = 0.028. Hypertension, hyperlipidemia, and osteoarthritis showed no effect. See Table 5, Model 3.

###### Model 4: Mental comorbidities

The past or present occurrence of anxiety syndrome significantly predicted bothersome tinnitus, Nagelkerke *R*^2^ = 0.014. Depression, panic, and social anxiety showed no effect. See Table 5, Model 4.

##### Model 5: Multivariable adjusted model

In the final regression analysis, all significant predictors from Models 1 to 4 were included in the same model for multivariable adjustment (for women and men, respectively). For both genders, higher age, somewhat reduced and very reduced hearing ability, hearing-related difficulties in social situations, and poor sleep quality were significant predictors of bothersome tinnitus. Additionally, cardiovascular disease and epilepsy were significant predictors for women, and education and anxiety syndrome for men. Regarding the level of education, secondary school, university, and “other” were associated with a lower risk of bothersome tinnitus compared with primary school. Model summary can be found in Table 6; female participants: Nagelkerke *R*^2^ = 0.153; male participants: *R*^2^ = 0.167.

**TABLE 6:** Multivariable adjusted regression model for the prediction of bothersome tinnitus (Model 5).

						**95% CI**	
**Variable**	**β**	**SE β**	**Wald’s *X ^2^***	***p***	**OR**	**Lower**	**Upper**
	**Female participants**						

Age^∗^	0.013	0.007	3.86	0.049	1.013	1.000	1.027
Education							
Employment							
Self-rated hearing ability^∗∗∗^			38.42	<0.001			
Somewhat reduced^∗∗∗^	0.823	0.823	32.69	<0.001	2.277	1.717	3.018
Very reduced^∗∗∗^	1.431	0.319	20.12	<0.001	4.182	2.238	7.815
Hearing-related difficulties in social situations^∗∗∗^	0.834	0.177	22.15	<0.001	2.302	1.627	3.258
Poor sleep quality^∗^	0.122	0.060	4.08	0.043	1.129	1.004	1.271
Cardiovascular disease^∗^	0.480	0.237	4.11	0.043	1.616	1.016	2.568
Thyroid disease							
Fibromyalgia							
Chronic shoulder pain							
Osteoarthritis							
Epilepsy^∗^	1.059	0.457	5.36	0.021	2.883	1.176	7.067
Burnout							

	**Male participants**						

Age^∗∗^	0.017	0.005	11.20	0.001	1.017	1.007	1.027
Education^∗∗^			16.20	0.001			
Secondary school^∗∗^	−0.781	0.280	7.76	0.005	0.458	0.265	0.793
University^∗∗∗^	−1.059	0.271	15.23	<0.001	0.347	0.204	0.590
Other^∗∗^	−0.899	0.329	7.46	0.006	0.407	0.214	0.776
Self-rated hearing ability^∗∗∗^			52.96	<0.001			
Somewhat reduced^∗∗∗^	1.053	0.156	45.32	<0.001	2.865	2.109	3.893
Very reduced^∗∗∗^	1.872	0.357	27.50	<0.001	6.499	3.229	13.082
Hearing-related difficulties in social situations^∗∗^	0.635	0.211	9.06	0.003	1.887	1.248	2.852
Poor sleep quality^∗∗∗^	0.267	0.068	15.35	<0.001	1.306	1.143	1.493
Chronic shoulder pain							
Ménière’s disease							
Anxiety syndrome^∗^	0.464	0.216	4.62	0.032	1.590	1.042	2.427

*Only significant results are displayed. OR, odds ratio. The coefficients of education are contrasted with 9-year primary school; those of self-rated hearing ability are contrasted with good hearing. ^∗∗∗^*p* < 0.001, ^∗∗^*p* < 0.01, ^∗^*p* < 0.05.*

#### Gender-Moderated Logistic Regression Analysis (Comparison Between Genders)

Predictors identified by the logistic regression analysis within men and women (all predictors included in Model 5 for female and/or male participants) were further analyzed to investigate whether gender moderates their effects on bothersome tinnitus, that is, whether their effects on bothersome tinnitus are different for women and men. These analyses revealed main effects (gender-independent) of age, hearing ability, hearing-related difficulties in social situations, cardiovascular disease, epilepsy and burnout; and moderating effects of gender for education and anxiety syndrome, see Table 7. In female participants, the effect of education was non-significant (*p* = 0.826); in male participants, higher education levels were negatively related to the presence of bothersome vs. non-bothersome tinnitus: β = −0.374, SE = 0.090, OR = 0.688 [0.577, 0.821], *p* < 0.001. For anxiety syndrome, the effect was non-significant in female participants (*p* = 0.116); in male participants, anxiety syndrome was associated with an increase of odds of bothersome tinnitus: β = 0.729, SE = 0.183, OR = 2.072 [1.449, 2.964], *p* < 0.001. For all other variables (employment, sleep quality, thyroid disease, fibromyalgia, chronic shoulder pain, osteoarthritis, Ménière’s disease) neither main effects nor moderation effects by gender were present.

**TABLE 7:** Moderation analysis by gender for the prediction of bothersome tinnitus.

					**95% CI**	
	**β**	**SE β**	***p***	**OR**	**Lower**	**Upper**
**Main effects**						

Age^∗∗^	0.029	0.009	0.001	1.029	1.011	1.047
Self-rated hearing ability^∗∗∗^	1.039	0.220	<0.001	2.828	1.837	4.351
Hearing-related difficulties in social situations^∗∗∗^	1.589	0.316	<0.001	4.897	2.636	9.096
Cardiovascular disease^∗^	1.168	0.465	0.012	3.216	1.293	7.997
Epilepsy^∗^	3.020	1.310	0.021	20.481	1.571	267.040
Burnout^∗∗^	0.938	0.344	0.007	2.554	1.301	5.013

**Moderation effects**						

Education × Gender^∗∗^	−0.393	0.125	0.002	0.675	0.528	0.863
Anxiety syndrome × Gender^∗^	0.494	0.236	0.036	1.638	1.032	2.601

*Only significant results are displayed. OR, odds ratio. ^∗∗∗^*p* < 0.001, ^∗∗^*p* < 0.01, ^∗^*p* < 0.05.*

## Discussion

The current study investigated a range of potential risk factors for bothersome tinnitus in a large Swedish sample. Our results indicate that participants with bothersome tinnitus differ from those with non-bothersome tinnitus in several aspects. Higher age, reduced hearing ability, more hearing-related difficulties in social situations, the past or present occurrence of cardiovascular disease, epilepsy, and burnout were associated with bothersome tinnitus in both genders, whereas associations of low education and anxiety syndrome were only present in male participants.

### Clear Associations With Bothersome Tinnitus

The effects of age and hearing loss on bothersome tinnitus are in accordance with the findings of Kim et al. (2015). Relationships between cardiovascular diseases and severe tinnitus have been reported previously (Nondahl et al., 2002; Stobik et al., 2005; Park et al., 2014), however, the mechanisms linking cardiovascular health and tinnitus are poorly understood. In a recent study, tinnitus was found to be twice as prevalent in patients with epilepsy than control subjects, and the authors argue that tinnitus may be related to the disease itself as well as to its long-term drug treatment (Hamed and Oseilly, 2018). Moreover, both conditions are linked to cortical hyperexcitability (Chai et al., 2019). Our study suggests an association between epilepsy and severe tinnitus, however, the nature of this association remains to be established. Burnout, a syndrome resulting from chronic occupational stress, is not a diagnostic category in itself, but can be understood as a form of depression (Bianchi et al., 2015). Our finding of a relationship between bothersome tinnitus and burnout is consistent with the results of a cross-sectional study by Hébert et al. (2012), who report that emotional exhaustion (which is part of the burnout symptomatology) is a predictor of tinnitus severity. In addition, Herr et al. (2016) found that burnout mediates the effects of work-related stress (low organizational justice) on tinnitus. However, we only found an effect of burnout in the moderation analysis, but not in the multivariable adjusted regression analysis.

### Unclear Associations With Bothersome Tinnitus

Female and male participants with bothersome tinnitus differed from those with non-bothersome tinnitus regarding marital status and employment (in frequency analyses). Being separated, divorced or widowed, and being unemployed, in early retirement, or running an owned or part-owned company might constitute stress factors, which can trigger or increase adverse tinnitus effects (Henry et al., 2005). Stress and tinnitus annoyance have been linked in the literature (Park et al., 2014; Kim et al., 2015). Higher alcohol consumption frequencies in men with bothersome tinnitus (more often drinking alcohol four times a week) could reflect maladaptive coping strategies to reduce tinnitus-related distress, as drinking to cope with negative affect is a relatively common drinking motive (Cooper et al., 1995; Kuntsche et al., 2014; Mohr et al., 2018). However, these sociodemographic and lifestyle factors showed no significant influence in regression analyses. Previous studies found a higher risk of tinnitus in patients with sleep apnea (Koo and Hwang, 2017), as well as associations between sleep disturbances and severe tinnitus (Axelsson and Ringdahl, 1989; Izuhara et al., 2013). In our analyses, poor sleep quality was a significant predictor of bothersome tinnitus in regression analyses for both male and female participants (with adjustment for other relevant factors), but no main effect of sleep quality was present in the moderation analysis.

### Gender-Specific Associations With Bothersome Tinnitus

Lower levels of education and past or present comorbid anxiety syndrome were specifically related to bothersome tinnitus in men. Several studies report links between low education levels and tinnitus (Fujii et al., 2011; Kim et al., 2015; House et al., 2018), as well as a relationship between lower education levels and higher tinnitus impairment (Unterrainer et al., 2001). Moreover, low education levels in individuals with tinnitus seem associated with poorer quality of life (Jung et al., 2019). Yet other studies found no relationship between tinnitus and education (Michikawa et al., 2010; Gallus et al., 2015), and one study observed higher tinnitus severity in individuals with higher education levels (Hoekstra et al., 2014). Thus, the literature is not conclusive in this regard. In addition, the relationship between low education and severe tinnitus might be confounded by socioeconomic status, occupational factors (e.g., noise exposure), or reduced openness to or accessibility of psychological treatment approaches (Unterrainer et al., 2001; Jung et al., 2019). Some of these confounding influences could potentially be stronger for men. As we did not assess these factors, we cannot exclude confounding influences. Furthermore, women generally reported higher education levels in our sample than men, which limits the generalization of this result.

Comorbidity between tinnitus and anxiety disorders is high, and they might share underlying neurobiological mechanisms (Pattyn et al., 2016; Lin et al., 2018). Anxiety can be not only a predisposing factor for severe tinnitus, but also a consequence of it, which can in turn increase tinnitus-related distress and impede habituation (Henry et al., 2005; Pattyn et al., 2016). Thus, the link between anxiety and bothersome tinnitus is not surprising. However, the prevalence of most anxiety disorders is around twice as high in women as in men (Bandelow and Michaelis, 2015). Accordingly, previous studies found higher anxiety levels in women with constant tinnitus than men (Schlee et al., 2017), and also in our sample, anxiety syndrome was more frequent in women than in men (12.5 vs. 7.6%). This result might indicate that even though men are less likely to be affected by anxiety symptoms, for them the contribution of anxiety on tinnitus annoyance is particularly strong.

### Clinical Implications

The medical assessment of tinnitus patients should include screenings for comorbidities, especially cardiovascular disease and epilepsy, which must be considered in clinical management. Anxiety and burnout should also be routinely investigated, as psychological treatments that target cognitive-affective sequelae of bothersome tinnitus have great potential to improve tinnitus burden (Henry et al., 2005; Pattyn et al., 2016; Cima, 2018). Similarly, hearing loss and sleep disturbances need to be addressed in all patients. In men particularly, the use of alcohol should be considered as a possible maladaptive coping strategy. Although different factors might be important for the emergence and maintenance of tinnitus-related distress in women and men, treatment should always be tailored to the individual physical and mental situation – including stress factors, cognitive and emotional reactions, and coping behaviors – to help the individual to better accept and habituate to the tinnitus (Henry et al., 2005; Cima, 2018).

### Limitations

Some limitations must be considered. As the design of this study is cross-sectional, no information on the causality of relationships can be derived. For many factors, the relationships with bothersome tinnitus are most likely complex and bidirectional; for example, anxiety can precede, be caused by, or increase tinnitus-related distress (Henry et al., 2005; Pattyn et al., 2016). The temporal relation between comorbidities and bothersome tinnitus could not be determined in our data. The aim of this study was to identify potential risk factors and comorbidities of bothersome tinnitus in women and men, yet case-control and longitudinal cohort studies are needed to investigate the mechanisms behind these associations. Furthermore, the investigated risk factors are not independent and, in some cases, moderately correlated. We conducted multivariate regression models in order to adjust for intercorrelations between variables, however, not all potentially confounding variables could be controlled for. Another limitation is the fact that all information on the investigated conditions (including tinnitus) stems from self-report data with apparent validity and reliability limitations. Furthermore, most variables were assessed with single-item questions as opposed to well-constructed and validated psychometric instruments. Regarding tinnitus specifically, no information on duration, localization or other characteristics was available. The comparatively high tinnitus rate in our sample may have been influenced by the fact that the question used to assess tinnitus did not specify a minimum duration of the experience (e.g., at least 5 min) unlike many other studies (McCormack et al., 2016), and did not specify the tinnitus onset, thus combining acute and chronic tinnitus. In addition, the question confounded continuity and annoyance of tinnitus, as “non-bothersome tinnitus” was defined as occurring sometimes, and “bothersome tinnitus” was defined as occurring constantly. Yet studies indicate that the tinnitus-associated burden and functional impact of constant tinnitus are indeed higher than those of intermittent/occasional tinnitus (Schlee et al., 2017; Koops et al., 2019).

## Conclusion

In summary, the present study found general associations between bothersome tinnitus and higher age, reduced hearing ability, hearing-related difficulties in social situations, cardiovascular disease, epilepsy, and burnout. In men, low education levels and comorbid anxiety might exert specific influences in the emergence or maintenance of bothersome tinnitus. Yet the effects of low education, in particular, must be interpreted with caution because of possible confounding influences. These new findings obtained from a large general population sample add to the literature of gender differences in tinnitus and imply the need for medical as well as psychological screenings of affected individuals and personalization of clinical treatment pathways. Future studies should investigate the mechanisms behind these general and gender-specific associations with bothersome tinnitus.

## Data Availability Statement

The data analyzed in this study is subject to the following licenses/restrictions: Restrictions are based on the Swedish Act (2013:794) requiring that a valid ethical approval is obtained in Sweden. Requests to access these datasets should be directed to NP, nancy.pedersen@ki.se.

## Ethics Statement

The studies involving human participants were reviewed and approved by the Local Ethics Committee “Regionala etikprövningsnämnden” in Stockholm (2015/2129-31/1). Adult participants provided their written informed consent to participate in this study. For participants under the age of 18, written informed consent to participate in this study was provided by the participants’ legal guardian/next of kin.

## Author Contributions

BM, CC, NP, and BC devised the project. BM, BB, PB, and LB conceived the study. NP provided the data. BB and LB devised the analysis strategy. LB performed the statistical analysis and wrote the first draft of the manuscript. BM, BB, PB, CC, NP, and BC critically reviewed the manuscript. All authors contributed to the article and approved the submitted version.

## Conflict of Interest

CC is supported by the UK National Institute for Health Research (NIHR) Biomedical Research Centre but the views expressed herein are his own and do not represent those of NIHR nor the UK Department of Health and Social Care.

The remaining authors declare that the research was conducted in the absence of any commercial or financial relationships that could be construed as a potential conflict of interest.
