# Supplementary material for: Gender-Specific Risk Factors and Comorbidities of Bothersome Tinnitus
Source: Front Neurosci. 2020 Sep 23;14:706. doi: 10.3389/fnins.2020.00706 (PMC7539146; doi:10.3389/fnins.2020.00706)
Supplement: Supplementary file 1 [file Table_1.docx]

## Table S1.

## *Assessment of traumatic or stressful experiences.*

| SL1. Har något av följande hänt dig under din livstid? (Markera alla aktuella) / Has any of the following happened to you in your lifetime? (Select all that applies) | |
| --- | --- |
|  | - Egen skilsmässa eller separation / Your own divorce or separation |
|  | - Allvarliga ekonomiska problem (t.ex. inga pengar till mat) / Serious financial problems (e.g. no money for food) |
|  | - Blivit diskriminerad på ett mycket besvärande sätt / Been discriminated against in a very bothersome way |
|  | - Varit utsatt för mobbning / Been subjected to bullying |
|  | - Bevittnat en allvarlig olycka / Witnessed a serious accident |
|  | - Varit involverad i en allvarlig olycka / Been involved in a serious accident |
|  | - Varit med om en naturkatastrof (t.ex. tsunami, orkan, skogsbrand) / Been involved in natural disaster (e.g. tsunami, hurricane, forest fire) |
|  | - Åkt in i fängelse för ett brott / Went to prison for a crime |
|  | - Nära familjemedlem som åkt in i fängelse / Close family member went to prison |
|  | - Inget av dessa / None of these |
|  | - Vet ej / vill ej svara / Don't know / Don't want to respond |
| SL2. Har något av följande hänt dig under din livstid? (Markera alla aktuella) / Has any of the following happened to you in your lifetime? (Select all that applies) | |
|  | - En nära vän som dött / A close friend has died |
|  | - Allvarlig fysisk eller psykisk sjukdom / A serious physical or psychological disease |
|  | - En nära familjemedlem med allvarlig fysisk eller psykisk sjukdom / A close family member with serious physical or psychological disease |
|  | - En nära familjemedlem som dött (förutom barn) / A close family member has died (except children) |
|  | - Ett barn som dött / A child that died |
|  | - Blivit adopterad eller placerad i familjehem / fosterhem / Been adopted or placed into foster care |
|  | - Före 18 år varit med om en skilsmässa eller separation mellan dina föräldrar / Before 18 years experienced a divorce or separation between your parents |
|  | - Separerats från ditt barn mot din vilja / Been separated from your child against your will |
|  | - Före 18 år blivit vittne till misshandel mellan familjemedlemmar / Before 18 years witnessed physical abuse between family members |
|  | - Blivit fysiskt försummad (t.ex. inte fått mat) / Been physically neglected (e.g. not been fed) |
|  | - Blivit känslomässigt misshandlad eller försummad (t.ex. ofta fått skämmas) / Been emotionally abused or neglected (e.g. often been embarrassed) |
|  | - Inget av dessa / None of these |
|  | - Vet ej / vill ej svara / Don't know / Don't want to respond |
| SL3. Har du upplevt något av följande? (Markera alla aktuella) / Have you experienced any of the following? (Select all that applies) | |
|  | - Varit vittne till ett rån eller personrån / Witnessed a robbery or mugging |
|  | - Varit utsatt för ett rån eller personrån / Been the victim of a robbery or mugging |
|  | - Blivit förföljd, hotad att bli dödad eller allvarligt skadad / Been stalked, threatened to be killed or seriously injured |
|  | - Före 18 år varit utsatt (ej sexuellt) eller misshandlad av någon du kände / Before 18 years been beaten by someone you knew |
|  | - Som vuxen varit utsatt (ej sexuellt) eller misshandlad av någon du kände / As an adult been beaten by someone you knew |
|  | - Besvärats eller trakasserats med sexuella tillmälen / Been troubled or harassed with sexual insults |
|  | - Före 18 år blivit utsatt för sexuellt ofredande eller tvingats till sexuella handlingar mot din vilja / Before 18 years been subject for sexual molestation or forced to sexual actions against your will |
|  | - Som vuxen blivit utsatt för sexuellt ofredande eller tvingats till sexuella handlingar / As an adult been sexually molested or forced to sexual actions |
|  | - Före 18 år tvingats att ha sex / Before 18 years been forced to sex |
|  | - Tvingats att ha sex som vuxen / Been forced to have sex as an adult |
|  | - Haft sex i utbyte mot pengar eller droger / Had sex in exchange for money or drugs |
|  | - Inget av dessa / None of these |
|  | - Vet ej / vill ej svara / Don't know / Don't want to respond |
| SL4. Har du varit allvarligt upprörd över att någon närstående varit utsatt för någon av dessa stressande livshändelser? / Have you been seriously upset about that anyone close has been subjected to any of these stressful life events? | |
|  | - Ja / Yes |
|  | - Nej / No |
|  | - Vet ej / vill ej svara / Don't know / Don't want to respond |
