# Supplementary figures and images for: Gender-Specific Risk Factors and Comorbidities of Bothersome Tinnitus
Source: Front Neurosci. 2020 Sep 23;14:706. doi: 10.3389/fnins.2020.00706 (PMC7539146; doi:10.3389/fnins.2020.00706)

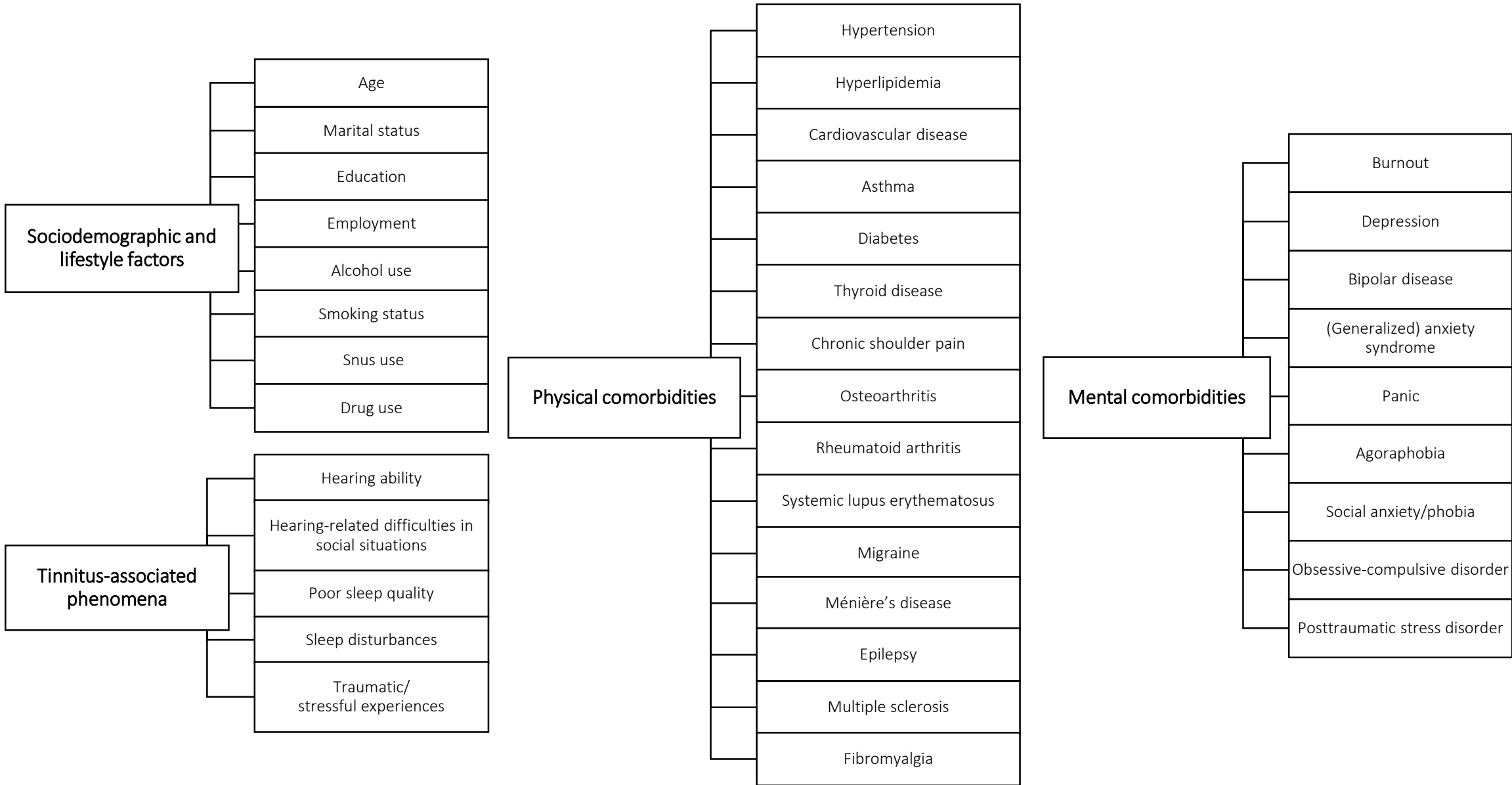

Supplement: Supplementary file 2 [file Image_1.pdf]
